# National trends in rheumatoid arthritis and osteoarthritis prevalence in South Korea, 1998–2021

**DOI:** 10.1038/s41598-023-46279-6

**Published:** 2023-11-09

**Authors:** Jaeyu Park, Myeongcheol Lee, Hojae Lee, Hyeon Jin Kim, Rosie Kwon, Hwi Yang, Seung Won Lee, Sunyoung Kim, Masoud Rahmati, Ai Koyanagi, Lee Smith, Min Seo Kim, Louis Jacob, Guillermo Felipe López Sánchez, Dragioti Elena, Jae Il Shin, Sang Youl Rhee, Myung Chul Yoo, Dong Keon Yon

**Affiliations:** 1 Center for Digital Health, Medical Science Research Institute, Kyung Hee University Medical Center, Kyung Hee University College of Medicine, Seoul, South Korea; 2 Department of Regulatory Science, Kyung Hee University, Seoul, South Korea; 3 Department of Precision Medicine, Sungkyunkwan University School of Medicine, Suwon, South Korea; 4 Department of Family Medicine, Kyung Hee University Medical Center, Kyung Hee University College of Medicine, Seoul, South Korea; 5 Department of Physical Education and Sport Sciences, Faculty of Literature and Human Sciences, Lorestan University, Khoramabad, Iran; 6 Department of Physical Education and Sport Sciences, Faculty of Literature and Humanities, Vali-E-Asr University of Rafsanjan, Rafsanjan, Iran; 7 Research and Development Unit, Parc Sanitari Sant Joan de Deu, Barcelona, Spain; 8 Centre for Health, Performance and Wellbeing, Anglia Ruskin University, Cambridge, UK; 9 Cardiovascular Disease Initiative, Broad Institute of MIT and Harvard, Cambridge, MA USA; 10 Physical Medicine and Rehabilitation Department, Lariboisière-Fernand Widal Hospital, AP-HP, Paris, France; 11 Epidemiology of Ageing and Neurodegenerative Diseases, Université Paris-Cité, Paris, France; 12 Division of Preventive Medicine and Public Health, Department of Public Health Sciences, School of Medicine, University of Murcia, Murcia, Spain; 13 Pain and Rehabilitation Centre, and Department of Medical and Health Sciences, Linköping University, Linköping, Sweden; 14 Research Laboratory Psychology of Patients, Families, and Health Professionals, Department of Nursing, School of Health Sciences, University of Ioannina, Ioannina, Greece; 15 Department of Pediatrics, Yonsei University College of Medicine, Seoul, South Korea; 16 Department of Endocrinology and Metabolism, Kyung Hee University School of Medicine, Seoul, South Korea; 17 Department of Rehabilitation Medicine, Kyung Hee University Medical Center, Kyung Hee University College of Medicine, 23 Kyungheedae-Ro, Dongdaemun-Gu, Seoul, 02447 South Korea; 18 Department of Pediatrics, Kyung Hee University Medical Center, Kyung Hee University College of Medicine, 23 Kyungheedae-Ro, Dongdaemun-Gu, Seoul, 02447 South Korea

**Keywords:** Rheumatoid arthritis, Osteoarthritis

## Abstract

Studies on the trends in the prevalence of rheumatoid arthritis (RA) and osteoarthritis (OA) are limited, particularly during the COVID-19 pandemic. This study aimed to analyze the temporal trend of RA and OA in South Korean adults from 1998 to 2021, including the COVID-19 pandemic period. The Korea National Health and Nutrition Examination Survey (KNHANES) data on adults aged ≥ 19 years were analyzed to investigate the prevalence of RA and OA from 1998 to 2021. The prevalence trends were compared by the years, and β_diff_ (β difference) was calculated. Odds ratios (ORs) were computed for each disease to examine changes in disease prevalence before and during the pandemic in order to determine the impact of the pandemic on disease prevalence. Among 163,221 Korean adults, the prevalence of RA and OA showed a steady decrease from 2005 (RA: from 1.91% in 2005–2007 to 1.55% in 2016–2019 and OA: from 9.75% in 2005–2007 to 8.27% in 2016–2019), but there was a slight increased after the onset of the COVID-19 pandemic (RA: from 1.23% in 2020 to 1.36% in 2021 and OA: from 8.04% in 2020 to 8.27% in 2021). Vulnerable groups, including participants aged ≥ 60 years (versus 19–60 years, ratio of ORs: 1.222; 95% CI 1.011–1.477), urban residents (ratio of ORs: 1.289; 95% CI 1.007–1.650), and participants with higher education level (ratio of ORs: 1.360; 95% CI 1.119–1.653) showed higher ORs of OA, whereas no particularly vulnerable population was observed for RA. Our findings provide an insight into the long-term trends of RA and OA among adult population and highlight a novel perspective on the impact of COVID-19 on disease prevalence.

## Introduction

Previous studies have suggested that the global prevalence rate of rheumatoid arthritis (RA) has remained consistent among decades^1^. However, in South Korea, several studies have reported a decrease in the prevalence of RA before the COVID-19 pandemic, followed by an increase during the pandemic^2^. They only provided information on RA prevalence from 2016 to 2020, where the impact of the pandemic to RA has not been reviewed. For this reason, we conducted a study on the prevalence trends of RA and OA between 1998 and 2021. Similar trends have been observed in the prevalence rate of osteoarthritis (OA) globally, with an increasing trend; however, in South Korea, it decreased before the pandemic and increased during the pandemic^3,4^.

Social distancing policy especially forcing to close the public gym facilities during the COVID-19 pandemic influenced domestic health concern^2,5,6^. Moreover, changes in physical activity, dietary habits, and mental health due to the COVID-19 restrictions may have influenced these prevalence rates^7^. However, further analysis and research are needed to determine the direct relationship between RA, OA, and the COVID-19 pandemic. Furthermore, some medications used to treat RA, such as corticosteroids and disease-modifying antirheumatic drugs, may suppress the immune system and increase the risk of infection^8^, thereby, making the patients more vulnerable to COVID-19 and its complications^8^. Therefore, it is crucial for patients with RA and COVID-19 to prioritize their health management^9^.

This study aimed to investigate the long-term trends in the prevalence of RA and OA using a representative large-scale dataset from 1998 to 2021. Additionally, we analyzed the specific social strata that were vulnerable during the COVID-19 period. Furthermore, our research findings may provide valuable information for developing policies for the treatment and prevention of these conditions following the COVID-19 pandemic.

## Methods

### Patient selection and data collection

This study utilized data from the Korea National Health and Nutrition Examination Survey (KNHANES) conducted between 1998 and 2021 by the Korea Centers for Disease Control and Prevention Agency (KDCA)^10,11^. The study population included adults aged ≥ 19 years, and the collected data included information on age, sex, residence, body mass index (BMI), education level, income, alcohol consumption, smoking status, and history of RA and OA^12^. We conducted our research focusing on the adult population, which ranges above 19 years in South Korea. A nationally representative sample of 163,221 participants was used to investigate the prevalence of RA and OA before and during the COVID-19 pandemic. The survey was conducted over 24 years and the number of participants surveyed in each year group was as follows: 51,515 in 1998–2001; 26,996 in 2005–2007; 20,070 in 2008–2010; 17,601 in 2011–2013; 17,129 in 2014–2016; 18,469 in 2017–2019; 5839 in 2020; and 5,602 in 2021.

The research protocol was approved by the Institutional Review Board of Kyung Hee University (KHUH 2022-06-042) and KDCA, and all participants provided written informed consent. Moreover, the KNHANES offers accessible public access to its data, which can be utilized as a valuable resource for diverse epidemiological investigations. This research adhered to the ethical guidelines established by relevant national and institutional review boards for human research and followed the 1975 Helsinki Declaration, as amended in 2008.

### Ascertainment of RA and OA

The objective of our study was to investigate the risk factors related to the two most common types of arthritis, RA and OA, over a period of 24 years from 1998 to 2021. To achieve our research objective, we surveyed a large sample of participants and asked them the question: “Have you ever been diagnosed with RA or OA by a doctor?” Based on their answers, we categorized the participants into three groups: RA, OA, and both^13^. We collected data on various potential risk factors associated with the development of RA and OA, such as age, sex, lifestyle habits, and socioeconomic status. We conducted statistical analyses to examine the associations between these risk factors and the development of RA and OA and to identify any patterns or trends that emerged over 24 years.

### Covariates

Covariates included age (19–29, 30–39, 40–49, 50–59, 60–69, 70–79, and ≥ 80 years), sex, region of residence (urban and rural)^14–16^, BMI group, household income (lowest, second, third, and highest quartile), education level (elementary school or lower, middle school, high school, and college or higher education), alcohol consumption (1–5 days/month, ≥ 6 days/month, and non-drinker), and smoking status (non-smoker, ex-smoker, and smoker). BMI was categorized into underweight (< 18.5 kg/m^2^), normal weight (18.5–22.9 kg/m^2^), overweight (23–25 kg/m^2^), and obese (≥ 25.0 kg/m^2^) according to the Asian-Pacific guidelines^17,18^.

### Statistical analyses

The results of this study were presented using qualitative data expressed as proportions or percentages. Weighted multivariate regression model analyses were conducted to compare the estimates of each related factor before and during the COVID-19 pandemic, with weighted odds ratios (ORs) with 95% confidence intervals (CIs)^19^. The prevalence of RA and OA was calculated using data from the KNHANES, spanning from 1998 to 2021, stratified by year group. Weighted complex sampling analysis was performed to ensure accurate estimation. Binomial or linear logistic regression models were used to compute the ORs with 95% CIs or β-coefficients with 95% CIs. To ensure robustness of the main findings, a stratification analysis was performed using variables such as sex, educational level, region of residence, and income in all the regression models. Furthermore, the ratio of ORs was calculated to estimate the interaction term of each risk factor and identify groups that were more vulnerable to the patient with RA and OA during the pandemic. Overall, this study aimed to provide a comprehensive analysis of the impact of the COVID-19 pandemic on the prevalence of RA and OA and to identify the factors that contribute to vulnerability to these conditions. The SAS software (version 9.4; SAS Institute, Cary, NC, USA) was used for statistical analyses, with a two-sided test, and a p-value ≤ 0.05 was considered statistically significant^19^.

## Results

We recruited 163,221 participants from 1998 to 2021 with the following distribution of characteristics: age (19–29 years, 18.18% [95% CI 17.82 to 18.53]; 30–39 years, 20.88% [20.47 to 21.29]; 40–49 years, 21.70% [21.35 to 22.06]; 50–59 years, 17.85% [17.56 to 18.14]; 60–69 years, 11.88% [11.64 to 12.12]; 70–79 years, 7.38% [7.19 to 7.58]; and ≥ 80 years, 2.13% [2.03 to 2.23]) and sex (male, 49.39% [49.14 to 49.64] and female, 50.61% [50.36 to 50.86]). These results are presented in Table 1.

**Table 1:** General characteristics of Korean adults, in the data obtained from the KNHANES from 1998 to 2021 (n = 163,221).

	Total	1998–2001	2005–2007	2008–2010	2011–2013	2014–2016	2017–2019	2020	2021
Overall, n	163,221	51,515	26,996	20,070	17,601	17,129	18,469	5839	5602
Age (years), weighted % (95% CI)									
19–29	18.18 (17.82 to 18.53)	19.90 (19.21 to 20.58)	21.34 (20.55 to 22.12)	18.80 (17.72 to 19.87)	17.59 (16.63 to 18.55)	16.78 (15.83 to 17.73)	16.51 (15.57 to 17.45)	16.19 (14.65 to 17.72)	15.91 (14.10 to 17.73)
30–39	20.88 (20.47 to 21.29)	25.85 (24.89 to 26.81)	24.20 (23.19 to 25.20)	22.23 (21.09 to 23.36)	20.55 (19.44 to 21.66)	19.06 (17.91 to 20.21)	17.75 (16.67 to 18.84)	16.77 (14.85 to 18.69)	16.32 (14.61 to 18.04)
40–49	21.70 (21.35 to 22.06)	23.47 (22.65 to 24.28)	23.22 (22.41 to 24.03)	22.55 (21.60 to 23.51)	21.95 (20.92 to 22.99)	21.13 (20.22 to 22.03)	20.18 (19.21 to 21.15)	19.35 (17.61 to 21.09)	18.99 (17.27 to 20.70)
50–59	17.85 (17.56 to 18.14)	13.87 (13.31 to 14.42)	14.69 (14.13 to 15.25)	17.11 (16.35 to 17.87)	18.85 (18.06 to 19.64)	20.02 (19.17 to 20.88)	20.13 (19.35 to 20.91)	20.03 (18.60 to 21.46)	19.78 (18.25 to 21.31)
60–69	11.88 (11.64 to 12.12)	11.29 (10.71 to 11.87)	10.24 (9.77 to 10.70)	10.45 (9.88 to 11.02)	10.83 (10.22 to 11.43)	12.06 (11.38 to 12.73)	13.62 (12.87 to 14.36)	15.04 (13.65 to 16.43)	16.06 (14.75 to 17.38)
70–79	7.38 (7.19 to 7.58)	5.35 (4.98 to 5.72)	5.85 (5.47 to 6.23)	6.92 (6.42 to 7.42)	7.94 (7.36 to 8.52)	8.27 (7.72 to 8.82)	8.41 (7.81 to 9.00)	8.85 (7.71 to 9.98)	8.93 (7.80 to 10.06)
≥ 80	2.13 (2.03 to 2.23)	0.28 (0.21 to 0.35)	0.47 (0.37 to 0.57)	1.95 (1.72 to 2.17)	2.28 (2.01 to 2.55)	2.69 (2.41 to 2.96)	3.40 (3.04 to 3.77)	3.78 (3.05 to 4.50)	4.01 (3.28 to 4.74)
Sex, weighted % (95% CI)									
Male	49.39 (49.14 to 49.64)	47.46 (47.10 to 47.82)	49.63 (49.16 to 50.10)	49.55 (48.89 to 50.21)	49.48 (48.76 to 50.20)	49.59 (48.87 to 50.31)	49.80 (49.07 to 50.53)	49.84 (48.73 to 50.95)	49.82 (48.52 to 51.12)
Female	50.61 (50.36 to 50.86)	52.54 (52.18 to 52.90)	50.37 (49.90 to 50.84)	50.45 (49.79 to 51.11)	50.52 (49.80 to 51.24)	50.41 (49.69 to 51.13)	50.20 (49.47 to 50.93)	50.16 (49.05 to 51.27)	50.18 (48.88 to 51.48)
Region of residence, weighted % (95% CI)									
Urban	82.15 (81.14 to 83.16)	80.86 (79.98 to 81.74)	81.29 (79.72 to 82.87)	80.07 (76.87 to 83.28)	80.73 (77.41 to 84.05)	83.26 (80.33 to 86.19)	84.68 (81.74 to 87.61)	84.57 (79.45 to 89.68)	83.80 (78.76 to 88.84)
Rural	17.85 (16.84 to 18.86)	19.14 (18.26 to 20.02)	18.71 (17.13 to 20.28)	19.93 (16.72 to 23.14)	19.27 (15.95 to 22.59)	16.74 (13.81 to 19.67)	15.32 (12.39 to 18.26)	15.43 (10.32 to 20.55)	16.20 (11.16 to 21.24)
BMI group, weighted % (95% CI)									
Underweight	3.52 (3.39 to 3.65)	1.12 (0.92 to 1.31)	1.44 (1.21 to 1.67)	4.69 (4.33 to 5.05)	4.79 (4.37 to 5.20)	4.31 (3.94 to 4.69)	3.92 (3.57 to 4.26)	4.13 (3.45 to 4.81)	4.17 (3.57 to 4.78)
Normal weight and overweight	49.31 (48.82 to 49.81)	15.90 (13.93 to 17.87)	20.53 (18.61 to 22.44)	63.19 (62.34 to 64.04)	62.22 (61.30 to 63.14)	61.47 (60.55 to 62.39)	60.81 (59.91 to 61.70)	56.40 (54.78 to 58.03)	57.52 (55.80 to 59.24)
Obesity	27.05 (26.68 to 27.41)	7.46 (6.50 to 8.43)	10.59 (9.53 to 11.64)	31.49 (30.65 to 32.33)	32.51 (31.57 to 33.44)	33.99 (33.04 to 34.94)	34.78 (33.90 to 35.67)	38.15 (36.60 to 39.71)	36.89 (35.11 to 38.68)
Unknown	20.12 (19.52 to 20.72)	75.52 (72.54 to 78.51)	67.44 (64.47 to 70.41)	0.63 (0.46 to 0.80)	0.48 (0.36 to 0.61)	0.23 (0.13 to 0.33)	0.49 (0.36 to 0.63)	1.31 (0.98 to 1.65)	1.41 (1.01 to 1.81)
Level of education, weighted % (95% CI)									
Elementary school or lower education	15.10 (14.77 to 15.43)	20.42 (19.49 to 21.35)	17.99 (17.16 to 18.82)	18.32 (17.22 to 19.41)	15.32 (14.30 to 16.34)	12.87 (12.00 to 13.74)	11.37 (10.46 to 12.28)	8.74 (7.32 to 10.16)	9.85 (8.31 to 11.40)
Middle school	9.49 (9.27 to 9.70)	12.26 (11.70 to 12.83)	10.67 (10.14 to 11.21)	10.49 (9.90 to 11.08)	9.56 (8.99 to 10.13)	8.43 (7.88 to 8.98)	7.94 (7.37 to 8.51)	7.16 (6.19 to 8.13)	7.14 (6.20 to 8.09)
High school	29.97 (29.58 to 30.36)	37.35 (36.39 to 38.31)	35.57 (34.67 to 36.48)	30.24 (29.22 to 31.27)	29.29 (28.19 to 30.39)	25.49 (24.48 to 26.50)	25.86 (24.87 to 26.84)	26.47 (24.65 to 28.29)	27.34 (25.50 to 29.18)
College or higher education	41.37 (40.79 to 41.94)	29.88 (28.63 to 31.13)	35.73 (34.52 to 36.95)	40.25 (38.64 to 41.86)	39.97 (38.40 to 41.54)	43.74 (42.12 to 45.37)	49.39 (47.66 to 51.11)	49.83 (46.59 to 53.08)	50.16 (47.12 to 53.21)
Unknown	4.08 (3.89 to 4.28)	0.09 (0.04 to 0.14)	0.03 (0.00 to 0.05)	0.70 (0.55 to 0.86)	5.86 (5.31 to 6.41)	9.47 (8.68 to 10.26)	5.45 (4.87 to 6.03)	7.80 (6.51 to 9.10)	5.50 (4.53 to 6.46)
Household income, weighted % (95% CI)									
Lowest quartile	16.99 (16.57 to 17.41)	22.79 (21.52 to 24.05)	18.02 (17.01 to 19.03)	16.81 (15.71 to 17.90)	16.04 (14.88 to 17.21)	15.76 (14.61 to 16.91)	15.67 (14.53 to 16.82)	15.08 (12.88 to 17.29)	14.11 (12.10 to 16.12)
Second quartile	24.86 (24.41 to 25.31)	24.49 (23.45 to 25.53)	25.80 (24.78 to 26.83)	25.45 (24.22 to 26.69)	27.01 (25.70 to 28.33)	23.79 (22.55 to 25.03)	24.30 (23.10 to 25.50)	22.10 (19.96 to 24.24)	22.79 (20.72 to 24.87)
Third quartile	28.55 (28.10 to 29.00)	25.43 (24.45 to 26.42)	28.50 (27.54 to 29.47)	28.76 (27.58 to 29.93)	28.58 (27.38 to 29.77)	29.91 (28.55 to 31.27)	28.52 (27.39 to 29.65)	29.24 (27.12 to 31.37)	30.07 (27.91 to 32.22)
Highest quartile	29.60 (28.98 to 30.23)	27.29 (25.84 to 28.74)	27.67 (26.28 to 29.07)	28.99 (27.31 to 30.66)	28.37 (26.76 to 29.97)	30.54 (28.68 to 32.39)	31.50 (29.79 to 33.22)	33.57 (30.09 to 37.05)	33.03 (29.29 to 36.77)
Smoking status, weighted % (95% CI)									
Smoker	19.26 (18.90 to 19.63)	9.23 (8.07 to 10.39)	11.42 (10.28 to 12.55)	27.20 (26.40 to 27.99)	24.02 (23.07 to 24.98)	21.69 (20.82 to 22.57)	20.73 (19.88 to 21.59)	19.30 (17.81 to 20.80)	18.27 (16.81 to 19.73)
Ex-smoker	16.52 (16.26 to 16.79)	2.63 (2.24 to 3.02)	7.42 (6.65 to 8.18)	19.83 (19.18 to 20.47)	18.55 (17.86 to 19.23)	19.83 (19.13 to 20.54)	22.27 (21.59 to 22.94)	23.82 (22.66 to 24.98)	25.02 (23.70 to 26.33)
Non-smoker	45.61 (45.10 to 46.12)	17.81 (15.63 to 19.98)	22.25 (20.23 to 24.28)	52.55 (51.80 to 53.31)	53.76 (52.86 to 54.65)	56.67 (55.78 to 57.57)	56.98 (56.13 to 57.84)	56.87 (55.43 to 58.32)	56.71 (55.05 to 58.37)
Unknown	18.60 (17.86 to 19.33)	70.34 (66.74 to 73.93)	58.91 (55.24 to 62.58)	0.42 (0.31 to 0.54)	3.67 (3.30 to 4.05)	1.81 (1.50 to 2.11)	0.01 (0.00 to 0.03)	0.00 (0.00 to 0.00)	0.00 (0.00 to 0.00)
Alcohol consumption, weighted % (95% CI)									
Non-drinker	12.08 (11.69 to 12.47)	14.91 (13.05 to 16.77)	13.99 (12.45 to 15.52)	11.76 (11.17 to 12.35)	12.18 (11.45 to 12.92)	12.21 (11.50 to 12.92)	9.75 (9.16 to 10.34)	9.82 (8.67 to 10.97)	9.70 (8.56 to 10.83)
1–5 days/month	51.21 (50.75 to 51.68)	11.11 (9.68 to 12.54)	18.66 (16.83 to 20.49)	63.98 (63.00 to 64.95)	62.24 (61.23 to 63.25)	63.42 (62.42 to 64.42)	67.00 (66.12 to 67.88)	68.13 (66.62 to 69.65)	70.46 (68.95 to 71.96)
6 ~ 30 days/month	18.11 (17.81 to 18.42)	3.64 (3.13 to 4.15)	8.45 (7.61 to 9.28)	23.84 (23.03 to 24.65)	21.90 (21.09 to 22.72)	22.57 (21.75 to 23.38)	23.23 (22.47 to 24.00)	22.05 (20.72 to 23.38)	19.85 (18.55 to 21.14)
Unknown	18.60 (17.86 to 19.33)	70.34 (66.74 to 73.93)	58.91 (55.24 to 62.58)	0.42 (0.31 to 0.54)	3.67 (3.30 to 4.05)	1.81 (1.50 to 2.11)	0.01 (0.00 to 0.03)	0.00 (0.00 to 0.00)	0.00 (0.00 to 0.00)

*BMI* body mass index, *CI* confidence interval, *KNHANES* Korea National Health and Nutrition Examination Survey.

*According to the Asian-Pacific guidelines, the BMI is divided into four groups: underweight (< 18.5 kg/m^2^), normal (18.5–22.9 kg/m^2^), overweight (23–24.9 kg/m^2^), and obese (≥ 25 kg/m^2^).

Table 2 illustrate the prevalence of RA, OA between pre-pandemic and during the pandemic. The prevalence of RA and OA showed a U-curve between pre-pandemic and during the pandemic (RA: from 1.46% [95% CI 1.38 to 1.54] in 2005–2019; 1.23% [0.92 to 1.54] in 2020; and 1.36% [1.02 to 1.69] in 2021, OA: from 7.68% [7.49 to 7.88] in 2005–2019; 8.04% [7.18 to 8.89] in 2020; and 8.27% [7.35 to 9.19] in 2021) (Table S1).

**Table 2:** National trends of the prevalence of RA and OA before and during the COVID-19 pandemic, weighted % (95% CI), in the data obtained from the KNHANES.

Year	Pre-pandemic (2005–2019)	During the pandemic	
		2020	2021
RA			
Overall	1.46 (1.38 to 1.54)	1.23 (0.92 to 1.54)	1.36 (1.02 to 1.69)
Age group			
Age (19–60 years)	0.95 (0.87 to 1.02)	0.56 (0.27 to 0.85)	0.73 (0.40 to 1.06)
Age (≥ 60 years)	3.40 (3.17 to 3.62)	2.98 (2.20 to 3.77)	2.89 (2.10 to 3.68)
Sex			
Male	0.74 (0.65 to 0.83)	0.53 (0.28 to 0.78)	0.82 (0.46 to 1.19)
Female	2.16 (2.04 to 2.28)	1.92 (1.34 to 2.51)	1.89 (1.41 to 2.36)
Region of residence			
Urban	1.37 (1.29 to 1.46)	1.21 (0.86 to 1.56)	1.22 (0.86 to 1.59)
Rural	1.85 (1.66 to 2.04)	1.32 (0.71 to 1.93)	2.05 (1.16 to 2.94)
BMI group			
Underweight	1.56 (1.11 to 2.01)	0.42 (0.00 to 1.02)	2.74 (0.47 to 5.01)
Normal weight and overweight	1.55 (1.44 to 1.66)	1.27 (0.84 to 1.70)	1.38 (0.95 to 1.82)
Obesity	1.61 (1.45 to 1.76)	1.17 (0.75 to 1.59)	1.11 (0.69 to 1.53)
Education			
High school or lower education	2.15 (2.03 to 2.27)	2.14 (1.57 to 2.71)	2.14 (1.61 to 2.67)
College or higher education	0.65 (0.56 to 0.74)	0.57 (0.26 to 0.87)	0.81 (0.38 to 1.25)
Income			
Income (lowest-second quartile)	1.95 (1.83 to 2.08)	1.94 (1.34 to 2.54)	1.82 (1.26 to 2.38)
Income (third-highest quartile)	1.10 (1.01 to 1.19)	0.81 (0.50 to 1.11)	1.09 (0.71 to 1.46)
OA			
Overall	7.68 (7.49 to 7.88)	8.04 (7.18 to 8.89)	8.27 (7.35 to 9.19)
Age group			
Age (19–60 years)	3.29 (3.15 to 3.43)	2.35 (1.80 to 2.90)	2.79 (2.19 to 3.39)
Age (≥ 60 years)	24.36 (23.80 to 24.92)	22.91 (20.86 to 24.97)	21.69 (19.53 to 23.85)
Sex			
Male	3.27 (3.10 to 3.44)	3.63 (2.86 to 4.40)	4.07 (3.22 to 4.92)
Female	11.99 (11.68 to 12.30)	12.42 (10.99 to 13.85)	12.44 (11.01 to 13.86)
Region of residence			
Urban	6.93 (6.73 to 7.14)	7.81 (6.89 to 8.74)	7.58 (6.65 to 8.52)
Rural	11.09 (10.49 to 11.70)	9.26 (6.58 to 11.94)	11.81 (9.18 to 14.44)
BMI group			
Underweight	2.84 (2.31 to 3.37)	3.07 (0.69 to 5.45)	2.96 (1.10 to 4.82)
Normal weight and overweight	6.86 (6.61 to 7.12)	7.33 (6.32 to 8.34)	7.23 (6.22 to 8.23)
Obesity	11.28 (10.87 to 11.70)	9.34 (7.94 to 10.74)	10.14 (8.64 to 11.63)
Education			
High school or lower education	12.56 (12.26 to 12.86)	15.87 (14.32 to 17.41)	14.75 (13.08 to 16.42)
College or higher education	1.77 (1.63 to 1.91)	2.42 (1.76 to 3.08)	3.36 (2.65 to 4.06)
Income			
Income (lowest-second quartile)	11.86 (11.52 to 12.20)	13.31 (11.74 to 14.88)	14.23 (12.41 to 16.05)
Income (third-highest quartile)	4.64 (4.45 to 4.82)	4.92 (4.12 to 5.71)	4.78 (4.00 to 5.56)

*CI* confidence interval, *KNHANES* Korea National Health and Nutrition Examination Survey, *OA* osteoarthritis, *OR* odds ratio, *RA* rheumatoid arthritis.

*Prevalence of RA, OA were available from 2005.

**Figure 1:**
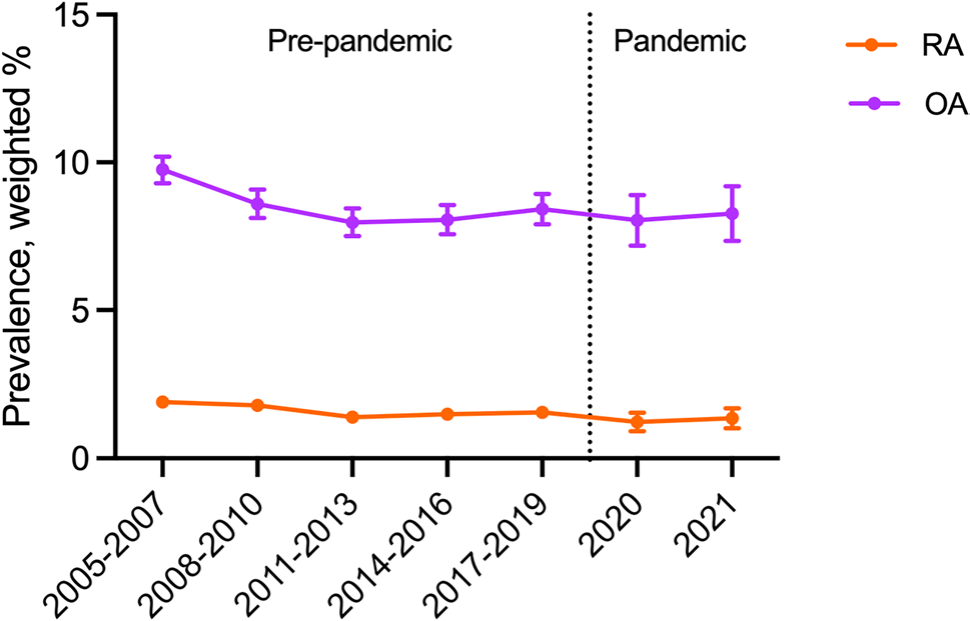
Seventeen-year trends in the prevalence of rheumatoid arthritis and osteoarthritis in South Korea, 2005–2021. *OA* osteoarthritis, *RA* rheumatoid arthritis.

Table 3 and Fig. 1 show the national trends in the prevalence of overall RA and OA, as well as the prevalence of RA and OA separately, along with the β-coefficients of the ORs before and during the COVID-19 pandemic. A statistically significant decrease in the weighted prevalence of RA was observed pre-pandemic (2005–2007, 1.91% [95% CI 1.72 to 2.10]; 2008–2010, 1.79% [1.59 to 1.99]; 2011–2013, 1.40% [1.23 to 1.58]; 2014–2016, 1.49% [1.31 to 1.67]; 2017–2019, 1.55% [1.36 to 1.75]; and 2020, 1.23% [0.92 to 1.54]), whereas a slight increase was observed during the pandemic (2020, 1.23% [0.92 to 1.54] and 2021, 1.36% [1.02 to 1.69]). Similarly, decrease in the weighted prevalence of OA was observed before the pandemic (2005–2007, 9.75% [9.29 to 10.20]; 2008–2010, 8.60% [8.12 to 9.08]; 2011–2013, 7.97% [7.51 to 8.44]; 2014–2016, 8.06% [7.57 to 8.56]; 2017–2019, 8.42% [7.91 to 8.93]. A slight increase was observed during the pandemic (2020, 8.04% [7.18 to 8.89] and 2021, 8.27% [7.35 to 9.19]). Similar patterns and trends were observed for the prevalence of OA and RA stratified by age, sex, region of residence, education level, and household income (Table S2). While investigating the weighted OR in 2021 with respect to that of 2020 (reference), there was a surge regarding RA among underweight group (OR, 6.62 [1.28 to 34.36]).

**Table 3:** National trend of the RA and OA prevalence and β-coefficients of the odds ratios before and during the COVID-19 pandemic, weighted % (95% CI), in the data obtained from the KNHANES.

Year	Pre-pandemic					During the pandemic		Trends in the pre-pandemic era, β (95% CI)	Trends in the pandemic era, β (95% CI)	β_diff_ between 2005–2019 and 2019–2021 (95% CI)	Weighted odds of before and during the pandemic, OR (95% CI)	
	2005–2007	2008–2010	2011–2013	2014–2016	2017–2019	2020	2021				2020 versus 2017–2019 (reference)	2021 versus 2020 (reference)
RA												
Overall	1.91 (1.72 to 2.10)	1.79 (1.59 to 1.99)	1.40 (1.23 to 1.58)	1.49 (1.31 to 1.67)	1.55 (1.36 to 1.75)	1.23 (0.92 to 1.54)	1.36 (1.02 to 1.69)	**− 0.097 (− 0.158 to − 0.036)**	− 0.098 (− 0.293 to 0.097)	0.000 (− 0.205 to 0.204)	0.79 (0.59 to 1.05)	1.11 (0.77 to 1.59)
Age group												
Age (19–60 years)	1.43 (1.24 to 1.62)	1.19 (0.99 to 1.39)	0.91 (0.73 to 1.09)	0.95 (0.76 to 1.15)	0.84 (0.65 to 1.02)	0.56 (0.27 to 0.85)	0.73 (0.40 to 1.06)	**− 0.141 (− 0.201 to − 0.082)**	− 0.054 (− 0.242 to 0.134)	0.087 (− 0.110 to 0.285)	0.66 (0.38 to 1.16)	1.31 (0.66 to 2.61)
Age (≥ 60 years)	4.35 (3.72 to 4.99)	4.28 (3.71 to 4.85)	3.24 (2.75 to 3.73)	3.29 (2.81 to 3.77)	3.66 (3.12 to 4.20)	2.98 (2.20 to 3.77)	2.89 (2.10 to 3.68)	**− 0.207 (− 0.389 to − 0.025)**	− 0.377 (− 0.856 to 0.102)	− 0.170 (− 0.683 to 0.342)	0.81 (0.59 to 1.11)	0.97 (0.66 to 1.43)
Sex												
Male	1.10 (0.90 to 1.31)	0.89 (0.66 to 1.12)	0.60 (0.41 to 0.80)	0.78 (0.57 to 0.99)	0.74 (0.52 to 0.96)	0.53 (0.28 to 0.78)	0.82 (0.46 to 1.19)	**− 0.079 (− 0.147 to − 0.011)**	0.041 (− 0.173 to 0.255)	0.120 (− 0.105 to 0.345)	0.71 (0.41 to 1.24)	1.56 (0.81 to 3.00)
Female	2.71 (2.41 to 3.01)	2.67 (2.35 to 2.99)	2.19 (1.88 to 2.49)	2.19 (1.89 to 2.48)	2.36 (2.05 to 2.66)	1.92 (1.34 to 2.51)	1.89 (1.41 to 2.36)	**− 0.114 (− 0.210 to − 0.019)**	− 0.235 (− 0.513 to 0.042)	− 0.121 (− 0.415 to 0.173)	0.81 (0.58 to 1.14)	0.98 (0.66 to 1.46)
Region of residence												
Urban	1.74 (1.53 to 1.94)	1.59 (1.38 to 1.81)	1.36 (1.17 to 1.55)	1.44 (1.24 to 1.63)	1.52 (1.30 to 1.73)	1.21 (0.86 to 1.56)	1.22 (0.86 to 1.59)	− 0.056 (− 0.123 to 0.011)	− 0.145 (− 0.357 to 0.067)	− 0.089 (− 0.312 to 0.133)	0.80 (0.57 to 1.11)	1.01 (0.66 to 1.55)
Rural	2.68 (2.17 to 3.19)	2.56 (2.08 to 3.05)	1.59 (1.14 to 2.03)	1.75 (1.31 to 2.20)	1.78 (1.31 to 2.24)	1.32 (0.71 to 1.93)	2.05 (1.16 to 2.94)	**− 0.265 (− 0.419 to − 0.111)**	**0.145 (− 0.349 to 0.638)**	0.409 (− 0.108 to 0.927)	0.74 (0.43 to 1.27)	1.56 (0.82 to 2.96)
BMI group												
Underweight	2.04 (0.54 to 3.54)	1.21 (0.49 to 1.94)	1.22 (0.29 to 2.15)	2.30 (1.08 to 3.51)	1.60 (0.61 to 2.58)	0.42 (0.00 to 1.02)	2.74 (0.47 to 5.01)	0.123 (− 0.224 to 0.471)	0.598 (− 0.633 to 1.828)	0.474 (− 0.804 to 1.753)	0.26 (0.06 to 1.23)	**6.62 (1.28 to 34.36)**
Normal weight and overweight	2.16 (1.73 to 2.59)	1.79 (1.54 to 2.04)	1.48 (1.25 to 1.71)	1.41 (1.20 to 1.62)	1.56 (1.30 to 1.82)	1.27 (0.84 to 1.70)	1.38 (0.95 to 1.82)	**− 0.116 (− 0.210 to − 0.021)**	− 0.090 (− 0.341 to 0.162)	0.026 (− 0.243 to 0.295)	0.81 (0.56 to 1.18)	1.09 (0.68 to 1.73)
Obesity	3.24 (2.46 to 4.01)	1.85 (1.49 to 2.21)	1.31 (1.02 to 1.61)	1.54 (1.19 to 1.89)	1.49 (1.19 to 1.80)	1.17 (0.75 to 1.59)	1.11 (0.69 to 1.53)	**− 0.229 (− 0.364 to − 0.094)**	− 0.189 (− 0.450 to 0.073)	0.041 (− 0.254 to 0.335)	0.78 (0.51 to 1.20)	0.95 (0.55 to 1.63)
Education												
High school or lower education	2.62 (2.33 to 2.90)	2.50 (2.21 to 2.79)	2.15 (1.87 to 2.43)	2.39 (2.07 to 2.71)	2.65 (2.29 to 3.01)	2.14 (1.57 to 2.71)	2.14 (1.61 to 2.67)	− 0.009 (− 0.110 to 0.092)	− 0.256 (− 0.580 to 0.067)	− 0.247 (− 0.586 to 0.092)	0.80 (0.59 to 1.10)	1.00 (0.68 to 1.46)
College or higher education	0.65 (0.48 to 0.82)	0.77 (0.53 to 1.02)	0.58 (0.36 to 0.80)	0.78 (0.56 to 1.00)	0.70 (0.51 to 0.89)	0.57 (0.26 to 0.87)	0.81 (0.38 to 1.25)	0.010 (− 0.053 to 0.072)	0.060 (− 0.176 to 0.297)	0.051 (− 0.194 to 0.295)	0.82 (0.45 to 1.48)	1.44 (0.67 to 3.07)
Income												
Income (lowest-second quartile)	2.71 (2.39 to 3.02)	2.27 (1.95 to 2.58)	1.86 (1.56 to 2.17)	2.01 (1.68 to 2.34)	2.19 (1.87 to 2.51)	1.94 (1.34 to 2.54)	1.82 (1.26 to 2.38)	**−** **0.125 (− 0.226 to − 0.024)**	− 0.189 (− 0.510 to 0.133)	− 0.064 (− 0.401 to 0.273)	0.88 (0.62 to 1.25)	0.94 (0.60 to 1.46)
Income (third-highest quartile)	1.29 (1.09 to 1.49)	1.44 (1.19 to 1.68)	1.06 (0.84 to 1.27)	1.15 (0.94 to 1.36)	1.13 (0.90 to 1.36)	0.81 (0.50 to 1.11)	1.09 (0.71 to 1.46)	− 0.060 (− 0.130 to 0.009)	− 0.017 (− 0.237 to 0.203)	0.043 (− 0.187 to 0.274)	0.71 (0.46 to 1.09)	1.35 (0.81 to 2.27)
OA												
Overall	9.75 (9.29 to 10.20)	8.60 (8.12 to 9.08)	7.97 (7.51 to 8.44)	8.06 (7.57 to 8.56)	8.42 (7.91 to 8.93)	8.04 (7.18 to 8.89)	8.27 (7.35 to 9.19)	**− 0.301 (− 0.457 to − 0.146)**	− 0.075 (− 0.610 to 0.460)	0.227 (− 0.331 to 0.784)	0.95 (0.83 to 1.10)	1.03 (0.87 to 1.23)
Age group												
Age (19–60 years)	4.83 (4.48 to 5.17)	4.21 (3.84 to 4.58)	3.28 (2.92 to 3.64)	3.27 (2.91 to 3.63)	2.89 (2.56 to 3.23)	2.35 (1.80 to 2.90)	2.79 (2.19 to 3.39)	**−** **0.478 (− 0.586 to − 0.369)**	− 0.056 (− 0.391 to 0.279)	**0.422 (0.069 to 0.774)**	0.81 (0.62 to 1.05)	1.19 (0.86 to 1.65)
Age (≥ 60 years)	34.53 (32.97 to 36.08)	26.96 (25.65 to 28.27)	25.57 (24.25 to 26.90)	24.11 (22.83 to 25.38)	24.63 (23.40 to 25.86)	22.91 (20.86 to 24.97)	21.69 (19.53 to 23.85)	**− 2.011 (− 2.442 to − 1.580)**	**− 1.462 (− 2.728 to − 0.196)**	0.549 (− 0.789 to 1.886)	0.91 (0.79 to 1.04)	0.93 (0.78 to 1.11)
Sex												
Male	4.79 (4.39 to 5.18)	3.75 (3.32 to 4.18)	3.08 (2.66 to 3.49)	3.36 (2.91 to 3.81)	3.28 (2.89 to 3.67)	3.63 (2.86 to 4.40)	4.07 (3.22 to 4.92)	**−** **0.327 (− 0.455 to − 0.200)**	0.396 (− 0.067 to 0.859)	**0.723 (0.243 to 1.203)**	1.11 (0.86 to 1.43)	1.13 (0.83 to 1.53)
Female	14.63 (13.92 to 15.35)	13.36 (12.59 to 14.13)	12.77 (11.99 to 13.54)	12.69 (11.93 to 13.46)	13.52 (12.68 to 14.36)	12.42 (10.99 to 13.85)	12.44 (11.01 to 13.86)	**− 0.268 (− 0.517 to − 0.018)**	− 0.541 (− 1.391 to 0.310)	− 0.273 (− 1.159 to 0.613)	0.91 (0.77 to 1.06)	1.00 (0.82 to 1.22)
Region of residence												
Urban	8.26 (7.82 to 8.71)	7.74 (7.25 to 8.24)	7.21 (6.72 to 7.71)	7.52 (7.00 to 8.04)	7.75 (7.21 to 8.29)	7.81 (6.89 to 8.74)	7.58 (6.65 to 8.52)	− 0.112 (− 0.271 to 0.048)	− 0.083 (− 0.625 to 0.458)	0.028 (− 0.536 to 0.593)	1.01 (0.87 to 1.18)	0.97 (0.80 to 1.17)
Rural	16.20 (14.54 to 17.85)	12.05 (10.55 to 13.55)	11.15 (9.76 to 12.54)	10.75 (9.21 to 12.29)	12.14 (10.38 to 13.89)	9.26 (6.58 to 11.94)	11.81 (9.18 to 14.44)	**− 0.933 (− 1.447 to − 0.420)**	− 0.124 (− 1.742 to 1.495)	0.810 (− 0.888 to 2.508)	0.74 (0.51 to 1.06)	1.31 (0.86 to 2.00)
BMI group												
Underweight	6.21 (3.78 to 8.64)	2.52 (1.54 to 3.50)	2.04 (1.09 to 2.98)	2.97 (1.70 to 4.24)	3.31 (2.05 to 4.58)	3.07 (0.69 to 5.45)	2.96 (1.10 to 4.82)	− 0.112 (− 0.578 to 0.354)	− 0.176 (− 1.396 to 1.045)	− 0.064 (− 1.370 to 1.243)	0.92 (0.38 to 2.23)	0.96 (0.33 to 2.82)
Normal weight and overweight	8.43 (7.51 to 9.36)	7.03 (6.52 to 7.55)	6.87 (6.33 to 7.42)	6.68 (6.15 to 7.21)	7.35 (6.78 to 7.92)	7.33 (6.32 to 8.34)	7.23 (6.22 to 8.23)	− 0.089 (− 0.298 to 0.121)	− 0.062 (− 0.644 to 0.519)	0.026 (− 0.592 to 0.644)	1.00 (0.83 to 1.19)	0.99 (0.79 to 1.23)
Obesity	14.39 (12.85 to 15.92)	12.71 (11.77 to 13.65)	11.02 (10.14 to 11.90)	11.26 (10.35 to 12.17)	10.86 (10.03 to 11.68)	9.34 (7.94 to 10.74)	10.14 (8.64 to 11.63)	**− 0.679 (− 1.012 to − 0.346)**	− 0.342 (− 1.226 to 0.542)	0.337 (− 0.608 to 1.282)	0.85 (0.70 to 1.02)	1.10 (0.86 to 1.39)
Education												
High school or lower education	14.10 (13.45 to 14.74)	13.39 (12.68 to 14.10)	13.51 (12.74 to 14.27)	15.09 (14.21 to 15.96)	16.10 (15.22 to 16.98)	15.87 (14.32 to 17.41)	14.75 (13.08 to 16.42)	**0.548 (0.304 to 0.792)**	− 0.674 (− 1.619 to 0.272)	**− 1.222 (− 2.198 to − 0.245)**	0.98 (0.86 to 1.13)	0.92 (0.77 to 1.10)
College or higher education	1.93 (1.63 to 2.23)	1.70 (1.38 to 2.02)	1.57 (1.23 to 1.91)	2.01 (1.68 to 2.35)	2.20 (1.87 to 2.53)	2.42 (1.76 to 3.08)	3.36 (2.65 to 4.06)	**0.103 (0.001 to 0.205)**	**0.579 (0.186 to 0.972)**	**0.476 (0.070 to 0.882)**	1.10 (0.80 to 1.52)	1.40 (0.98 to 2.00)
Income												
Income (lowest-second quartile)	15.07 (14.28 to 15.85)	12.86 (12.05 to 13.67)	12.15 (11.35 to 12.95)	13.02 (12.05 to 13.99)	13.71 (12.80 to 14.62)	13.31 (11.74 to 14.88)	14.23 (12.41 to 16.05)	− 0.239 (− 0.511 to 0.032)	0.254 (− 0.754 to 1.262)	0.493 (− 0.550 to 1.537)	0.97 (0.82 to 1.14)	1.08 (0.88 to 1.34)
Income (third-highest quartile)	5.60 (5.20 to 5.99)	5.48 (5.02 to 5.94)	4.81 (4.34 to 5.29)	4.82 (4.37 to 5.28)	4.90 (4.44 to 5.35)	4.92 (4.12 to 5.71)	4.78 (4.00 to 5.56)	**− 0.199 (− 0.337 to −0.060)**	− 0.059 (− 0.510 to 0.392)	0.140 (− 0.332 to 0.611)	1.00 (0.83 to 1.22)	0.97 (0.77 to 1.23)

The numbers in bold indicate a significant difference (p < 0.05).

*BMI* body mass index, *CI* confidence interval, *KNHANES* Korea National Health and Nutrition Examination Survey, *OA* osteoarthritis, *OR* odds ratio, *RA* rheumatoid arthritis.

Table 4 shows the pandemic-related effects on vulnerable groups of patients with RA and OA. A statistically significant difference was observed among vulnerable individuals with OA as follows: participants ≥ 60 years old (ratio of ORs 1.222 [95% CI 1.011 to 1.477]), urban residents (ratio of ORs 1.289 [1.007 to 1.650]), and participants with a high level of education (ratio of ORs 1.360 [1.119 to 1.653]). In contrast, no significant difference in pandemic-related effects was observed among vulnerable individuals with RA (Table S3).

**Table 4:** Difference between pre- and during the COVID-19 pandemic by the ratio of ORs on OA and RA, weighted % (95% CI), in the data obtained from the KNHANES.

Variables		Overall (2005–2021)	Pre-COVID-19 pandemic (2005–2019)	COVID-19 pandemic (2020, 2021)	Ratio of OR (95% CI)
		Weighted OR (95%CI)	Weighted OR (95%CI)	Weighted OR (95%CI)	
RA					
Age	19–59	1.000 (reference)	1.000 (reference)	1.000 (reference)	1.000 (reference)
	≥ 60	**3.596 (3.248 to 3.980)**	**3.553 (3.197 to 3.948)**	**4.665 (3.145 to 6.919)**	1.313 (0.873 to 1.975)
Sex	Female	1.000 (reference)	1.000 (reference)	1.000 (reference)	1.000 (reference)
	Male	**0.335 (0.298 to 0.377)**	**0.334 (0.295 to 0.379)**	**0.348 (0.244 to 0.497)**	1.042 (0.715 to 1.519)
Region	Urban	1.000 (reference)	1.000 (reference)	1.000 (reference)	1.000 (reference)
	Rural	**1.433 (1.272 to 1.615)**	**1.424 (1.257 to 1.614)**	**1.502 (1.015 to 2.223)**	1.055 (0.699 to 1.592)
Education	High school or less	1.000 (reference)	1.000 (reference)	1.000 (reference)	1.000 (reference)
	College or more	**0.245 (0.214 to 0.280)**	**0.241 (0.209 to 0.278)**	**0.286 (0.192 to 0.427)**	1.187 (0.777 to 1.814)
Income	High	1.000 (reference)	1.000 (reference)	1.000 (reference)	1.000 (reference)
	Low	**1.885 (1.710 to 2.079)**	**1.864 (1.683 to 2.066)**	**2.004 (1.460 to 2.752)**	1.075 (0.770 to 1.500)
OA					
Age	19–59	1.000 (reference)	1.000 (reference)	1.000 (reference)	1.000 (reference)
	≥ 60	**8.852 (8.467 to 9.254)**	**8.839 (8.442 to 9.254)**	**10.799 (8.986 to 12.980)**	**1.222 (1.011 to 1.477)**
Sex	Female	1.000 (reference)	1.000 (reference)	1.000 (reference)	1.000 (reference)
	Male	**0.256 (0.244 to 0.268)**	**0.253 (0.241 to 0.265)**	**0.286 (0.243 to 0.337)**	1.130 (0.953 to 1.340)
Region	Rural	1.000 (reference)	1.000 (reference)	1.000 (reference)	1.000 (reference)
	Urban	**0.535 (0.501 to 0.571)**	**0.523 (0.489 to 0.560)**	**0.674 (0.531 to 0.854)**	**1.289 (1.007 to 1.650)**
Education	High school or less	1.000 (reference)	1.000 (reference)	1.000 (reference)	1.000 (reference)
	College or more	**0.117 (0.110 to 0.125)**	**0.111 (0.103 to 0.119)**	**0.151 (0.126 to 0.181)**	**1.360 (1.119 to 1.653)**
Income	High	1.000 (reference)	1.000 (reference)	1.000 (reference)	1.000 (reference)
	Low	**2.864 (2.742 to 2.992)**	**2.839 (2.712 to 2.971)**	**3.129 (2.689 to 3.641)**	1.102 (0.941 to 1.291)

The numbers in bold indicate a significant difference (p < 0.05).

*KNHANES* Korea National Health and Nutrition Examination Survey, *CI* confidence interval, *OA* osteoarthritis, *OR* odds ratio, *RA* rheumatoid arthritis.

## Discussion

### Key results

The present study examined trends in the prevalence of RA and OA over a 24-year period from 1998 to 2021 and evaluated the differences in prevalence before and during the COVID-19 pandemic (n = 163,221). The prevalence of RA and OA consistently declined before the onset of the pandemic, but there was a slight increased during the pandemic. However slight increasing prevalence during pandemic was not statistically significant. Notably, patients with OA had a significantly high prevalence among vulnerable groups, including individuals aged ≥ 60 years, urban residents, and those with a high education level. Therefore, these findings suggest policy researchers should develop personalized policy proposals to address the needs of these groups during the pandemic.

### Global epidemiology and mechanism

Previous studies have reported a global increase in the prevalence of RA and OA, before the COVID-19 pandemic^20^. However, in South Korea, the prevalence of both conditions has consistently declined, contrary to global trends^21^. Similar trends of decline in the prevalence of RA have been observed in Japan and Sweden as well^22^. A Japanese study found a correlation between a decrease in RA prevalence and changes in lifestyle factors, such as dietary habits and smoking, while a Swedish study has linked this decrease to improvements in medical accessibility for patients with RA^16,23^. Although we cannot accurately identify the specific reasons for the variation in RA prevalence across countries, we believe that factors, such as genetic, cultural disparities, and health level may have a significant impact^24^. In addition, increased BMI and muscle weakness are closely related to OA and RA, as these diseases are associated with factors that contribute to joint strain^25,26^.

During the COVID-19 pandemic, the social distancing policies and the closure of sports facilities significantly diminished the public health standards, which could generally explain the observed increase in the prevalence of RA and OA^27,28^.

RA is a complex disease with multiple factors contributing to its development, with genetics potentially being a significant factor^29^. Other studies have reported that numerous variables can affect the prevalence of RA^30^. Therefore, further research is warranted to investigate this matter thoroughly. An increase in the prevalence of OA and RA was the most prominent in the highly educated older adult population living in urban areas^31^. These individuals who were retired and had reduced health standards due to COVID-19 were most affected^32^. According to previous research, the domestic standard of physical activity among South Korean adult population exhibited a declining trend until 2017, followed by an upturn until 2019^33^. In 2020, confronting the pandemic, there was a significant decline in physical activity. This pattern closely aligns with the prevalence trends in RA and OA, which could substantiate our hypothesis.

This finding suggests that OA may occur when physical activity falls below a certain threshold, which warrants further investigation. It is crucial to recognize this issue at a national level and implement policies to encourage exercise among the older adults, prevent the occurrence of OA, and provide the appropriate treatment for those with the disease.

### Strengths and limitations

The present study is significant in utilizing population-based nationwide representative data to compare the trends and prevalence of RA and OA, both before and during the COVID-19 pandemic^34^. The use of national data is particularly noteworthy, as individual data collection during a pandemic could have been challenging. Furthermore, this study’s utilization of survey data collected from 1998 to 2021, spanning a period of 24 years, adds academic value to the research findings due to its long-term nature. However, this study has several limitations. First, data were collected only from Koreans, thus limiting generalizability of the results to other ethnic groups or countries. Further research with multiple ethnicities and different countries is necessary to examine the global impact of the COVID-19 pandemic^35^. Second, data on patients with separate covariate of RA and OA were only available from 2005–2021and information prior to 2005 was not available. Third, some variables in the study, such as height and weight, were self-reported, which may have introduced bias. To address this concern, the KDCA should include a question in the survey about when participants' heights and weights were last measured^36^. In addition, the diagnosis of RA and OA was self-reported, potentially resulting in a recall bias. Lastly, we substituted physical activity data from the previous study, since we were unable to conduct our own analysis of domestic physical activity levels.

## Conclusion

Our study identified long-term trends in the prevalence of RA and OA spanning 24-years from 1998 to 2021, especially focusing on the COVID-19 pandemic. The results showed a consistent decline in the prevalence of both RA and OA until 2016 followed by an oscillation until 2021, where significant drop occurred in 2019. Notably, OA exhibited a higher prevalence among vulnerable groups, such as individuals over 60 years of age, urban residents, and those with a high education level. When investigating the RA prevalence trend, there was no specific vulnerable population. This outcome will help government policy researchers devise personalized healthcare policies targeting the vulnerable groups. While our study did not find any evidence of a relationship between the COVID-19 pandemic and the prevalence of RA, additional follow-up studies is needed to further explore this topic.

### Supplementary Information

Supplementary Information.

## Data Availability

Data are available on reasonable request. Study protocol, statistical code: available from DKY (email: yonkkang@gmail.com). Data set: available from the Korea Disease Control and Prevention Agency (KDCA) through a data use agreement.
