# Supplementary material for: National trends in rheumatoid arthritis and osteoarthritis prevalence in South Korea, 1998–2021
Source: Sci Rep. 2023 Nov 9;13:19528. doi: 10.1038/s41598-023-46279-6 (PMC10636038; doi:10.1038/s41598-023-46279-6)
Supplement: Supplementary file 1 — Supplementary Information. [file 41598_2023_46279_MOESM1_ESM.pdf]

Original Article

National trends in rheumatoid arthritis and osteoarthritis prevalence in South Korea from, 1998 to 2021

Running title: **Rheumatoid arthritis and osteoarthritis in South Korea**

Jaeyu Park,[1,2] Myeongcheol Lee,[1,2] Hojae Lee,[1,2] Hyeon Jin Kim,[1,2] Rosie Kwon,[1,2] Hwi Yang,[1,2] Seung Won Lee,[3] Sunyoung Kim,[4] Masoud Rahmati,[5] Ai Koyanagi,[6,7] Lee Smith,[8] Min Seo Kim,[9] Louis Jacob,[6,7,10,11,] Guillermo F López Sánchez,[12] Dragioti Elena,[13,14] Jae Il Shin,[15] Sang Youl Rhee,[2,16] Myung Chul Yoo,[17*] Dong Keon Yon[1,2,18*]

***Corresponding authors**

Dong Keon Yon**, MD, FACAAI, FAAAAI**

Department of Pediatrics, Kyung Hee University Medical Center, Kyung Hee University College of Medicine, 23 Kyungheedae-ro, Dongdaemun-gu, Seoul 02447, South Korea

Email: yonkkang@gmail.com

Myung Chul Yoo**, MD, PhD**

Department of Rehabilitation Medicine, Kyung Hee University Medical Center, Kyung Hee University College of Medicine, 23 Kyungheedae-ro, Dongdaemun-gu, Seoul 02447, South Korea

Email: famousir@naver.com

Table S1. National trends of the prevalence of RA and OA before and during the COVID-19 pandemic, weighted % (95% CI), in the data obtained from the KNHANES

Year	Pre-pandemic (1998-2019)	During the pandemic	
		2020	2021
RA and OA			
Overall	9.74 (9.53 to 9.95)	8.91 (8.00 to 9.82)	9.27 (8.31 to 10.24)
Age group			
Age (19–60 years)	4.66 (4.50 to 4.83)	2.87 (2.28 to 3.46)	3.46 (2.81 to 4.11)
Age (≥60 years)	29.02 (28.44 to 29.60)	24.72 (22.58 to 26.85)	23.49 (21.27 to 25.71)
Sex			
Male	4.33 (4.14 to 4.52)	4.08 (3.31 to 4.86)	4.74 (3.83 to 5.64)
Female	15.02 (14.68 to 15.36)	13.71 (12.19 to 15.23)	13.77 (12.28 to 15.27)
Region of residence			
Urban	8.72 (8.50 to 8.94)	8.68 (7.69 to 9.66)	8.49 (7.50 to 9.48)

Rural	14.37 (13.67 to 15.06)	10.19 (7.34 to 13.05)	13.32 (10.61 to 16.02)
BMI group			
Underweight	4.38 (3.70 to 5.06)	3.49 (1.03 to 5.95)	4.78 (2.20 to 7.36)
Normal weight and overweight	8.40 (8.13 to 8.68)	8.29 (7.19 to 9.38)	8.24 (7.16 to 9.33)
Obesity	12.91 (12.47 to 13.35)	10.14 (8.66 to 11.61)	10.96 (9.43 to 12.49)
Education			
High school or lower education	15.75 (15.42 to 16.08)	17.27 (15.71 to 18.84)	16.33 (14.59 to 18.06)
College or higher education	2.50 (2.33 to 2.66)	2.94 (2.21 to 3.66)	3.96 (3.19 to 4.74)
Income			
Income (lowest-second quartile)	14.77 (14.40 to 15.15)	14.44 (12.78 to 16.09)	15.49 (13.66 to 17.32)
Income (third-highest quartile)	6.06 (5.86 to 6.27)	5.64 (4.82 to 6.46)	5.64 (4.81 to 6.46)

Abbreviations: CI, confidence interval; KNHANES, Korea National Health and Nutrition Examination Survey; OA, osteoarthritis; OR, odds ratio; RA, rheumatoid arthritis.

Table S2. National trend of the RA and OA prevalence and β-coefficients of the odds ratios before and during the COVID-19 pandemic, weighted % (95% CI), in the data obtained from the KNHANES

Year	Pre-pandemic						During the pandemic		Trends in the pre-pandemic era, β (95% CI)	Trends in the pandemic era, β (95% CI)	β_{diff} between 2005-2019 and 2019-2021 (95% CI)	Weighted odds of before and during the pandemic, OR (95% CI)	
	1998-2001	2005-2007	2008-2010	2011-2013	2014-2016	2017-2019	2020	2021				2020 versus 2017–2019 (reference)	2021 versus 2020 (reference)
RA and OA													
Overall	9.30 (8.79 to 9.81)	11.36 (10.87 to 11.85)	10.10 (9.58 to 10.61)	9.03 (8.53 to 9.53)	9.13 (8.61 to 9.65)	9.54 (9.00 to 10.07)	8.91 (8.00 to 9.82)	9.27 (8.31 to 10.24)	**-0.247 (-0.375 to -0.119)**	-0.131 (-0.695 to 0.433)	0.116 (-0.463 to 0.694)	0.93 (0.81 to 1.06)	1.05 (0.88 to 1.24)
Age group													

Age (19–60 years)	4.92 (4.56 to 5.28)	6.17 (5.78 to 6.55)	5.28 (4.88 to 5.69)	4.10 (3.68 to 4.52)	4.08 (3.68 to 4.48)	3.66 (3.29 to 4.03)	2.87 (2.28 to 3.46)	3.46 (2.81 to 4.11)	**-0.448 (-0.540 to -0.356)**	-0.101 (-0.470 to 0.267)	0.347 (-0.033 to 0.726)	**0.78 (0.61 to 0.98)**	1.22 (0.92 to 1.61)
Age (≥60 years)	30.80 (29.16 to 32.43)	37.52 (35.95 to 39.08)	30.20 (28.85 to 31.54)	27.52 (26.16 to 28.89)	26.04 (24.72 to 27.35)	26.77 (25.51 to 28.03)	24.72 (22.58 to 26.85)	23.49 (21.27 to 25.71)	**-1.759 (-2.108 to -1.410)**	**-1.630 (-2.924 to -0.335)**	0.129 (-1.212 to 1.470)	0.90 (0.79 to 1.03)	0.94 (0.79 to 1.11)
Sex													
Male	4.38 (3.96 to 4.80)	5.81 (5.36 to 6.25)	4.52 (4.04 to 5.00)	3.55 (3.10 to 4.01)	4.02 (3.53 to 4.50)	3.93 (3.51 to 4.36)	4.08 (3.31 to 4.86)	4.74 (3.83 to 5.64)	**-0.284 (-0.392 to -0.175)**	0.403 (-0.097 to 0.902)	**0.686 (0.175 to 1.197)**	1.04 (0.83 to 1.31)	1.17 (0.88 to 1.55)
Female	13.75 (12.98	16.83 (16.06	15.57 (14.75	14.39 (13.55	14.17 (13.36	15.10 (14.23	13.71 (12.19	13.77 (12.28	-0.158 (-0.360 to 0.044)	-0.659 (-1.547 to 0.230)	-0.501 (-1.412 to 0.410)	0.89 (0.77 to 1.04)	1.01 (0.83 to 1.22)

	to 14.53)	to 17.60)	to 16.40)	to 15.23)	to 14.97)	to 15.97)	to 15.23)	to 15.27)					
Region of residence													
Urban	7.20 (6.71 to 7.70)	9.80 (9.32 to 10.29)	9.09 (8.56 to 9.63)	8.23 (7.69 to 8.77)	8.58 (8.03 to 9.14)	8.84 (8.27 to 9.41)	8.68 (7.69 to 9.66)	8.49 (7.50 to 9.48)	0.020 (-0.112 to 0.153)	-0.175 (-0.748 to 0.398)	-0.195 (-0.784 to 0.393)	0.98 (0.85 to 1.14)	0.98 (0.81 to 1.17)
Rural	18.16 (16.45 to 19.88)	18.12 (16.40 to 19.84)	14.12 (12.46 to 15.77)	12.37 (10.83 to 13.91)	11.87 (10.30 to 13.44)	13.40 (11.60 to 15.21)	10.19 (7.34 to 13.05)	13.32 (10.61 to 16.02)	**-1.250 (-1.664 to -0.835)**	0.003 (-1.661 to 1.667)	1.252 (-0.462 to 2.967)	0.73 (0.52 to 1.04)	1.35 (0.91 to 2.02)
BMI group													
Underweight	4.62 (1.88	7.68 (4.95	3.67 (2.44	3.22 (1.92	4.87 (3.17	4.86 (3.29	3.49 (1.03	4.78 (2.20	0.012 (-0.482 to 0.507)	-0.019 (-1.589 to 1.552)	-0.031 (-1.677 to 1.615)	0.71 (0.32 to 1.56)	1.39 (0.55 to 3.53)

	to 7.36)	to 10.40)	to 4.90)	to 4.53)	to 6.58)	to 6.43)	to 5.95)	to 7.36)					
Normal weight and overweight	10.11 (8.70 to 11.53)	10.45 (9.44 to 11.46)	8.55 (7.99 to 9.11)	8.01 (7.42 to 8.61)	7.70 (7.13 to 8.27)	8.53 (7.92 to 9.14)	8.29 (7.19 to 9.38)	8.24 (7.16 to 9.33)	**-0.304 (-0.506 to -0.102)**	-0.143 (-0.770 to 0.484)	0.161 (-0.498 to 0.820)	0.97 (0.82 to 1.15)	0.99 (0.80 to 1.23)
Obesity	16.93 (14.99 to 18.87)	17.23 (15.48 to 18.98)	14.19 (13.19 to 15.18)	11.92 (10.99 to 12.84)	12.32 (11.38 to 13.25)	11.78 (10.93 to 12.64)	10.14 (8.66 to 11.61)	10.96 (9.43 to 12.49)	**-1.005 (-1.315 to -0.694)**	-0.392 (-1.298 to 0.515)	0.613 (-0.345 to 1.571)	0.85 (0.71 to 1.01)	1.09 (0.87 to 1.37)
Education													
High school or lower education	12.54 (11.86 to 13.21)	16.26 (15.58 to 16.95)	15.44 (14.68 to 16.20)	15.06 (14.24 to 15.88)	16.68 (15.76 to 17.60)	17.86 (16.95 to 18.77)	17.27 (15.71 to 18.84)	16.33 (14.59 to 18.06)	**0.714 (0.518 to 0.910)**	-0.768 (-1.753 to 0.217)	**-1.482 (-2.486 to -0.477)**	0.96 (0.84 to 1.09)	0.94 (0.79 to 1.11)

College or higher education	1.72 (1.40 to 2.04)	2.55 (2.22 to 2.89)	2.42 (2.01 to 2.83)	2.11 (1.70 to 2.51)	2.71 (2.33 to 3.10)	2.86 (2.49 to 3.23)	2.94 (2.21 to 3.66)	3.96 (3.19 to 4.74)	**0.150 (0.057 to 0.243)**	**0.556 (0.122 to 0.990)**	0.406 (-0.038 to 0.850)	1.03 (0.77 to 1.37)	1.36 (0.98 to 1.89)
Income													
Income (lowest-second quartile)	13.80 (12.97 to 14.64)	17.20 (16.37 to 18.03)	14.63 (13.75 to 15.51)	13.46 (12.60 to 14.33)	14.18 (13.18 to 15.18)	15.14 (14.20 to 16.08)	14.44 (12.78 to 16.09)	15.49 (13.66 to 17.32)	-0.165 (-0.385 to 0.054)	0.167 (-0.850 to 1.183)	0.332 (-0.708 to 1.372)	0.95 (0.80 to 1.11)	1.09 (0.89 to 1.33)
Income (third-highest quartile)	5.26 (4.83 to 5.70)	6.80 (6.36 to 7.24)	6.78 (6.25 to 7.30)	5.68 (5.15 to 6.21)	5.83 (5.34 to 6.32)	5.81 (5.32 to 6.29)	5.64 (4.82 to 6.46)	5.64 (4.81 to 6.46)	-0.111 (-0.228 to 0.007)	-0.085 (-0.564 to 0.394)	0.026 (-0.467 to 0.519)	0.97 (0.82 to 1.15)	1.00 (0.81 to 1.24)

Abbreviations: BMI, body mass index; CI, confidence interval; KNHANES, Korea National Health and Nutrition Examination Survey; OA, osteoarthritis; OR, odds ratio; RA, rheumatoid arthritis.

The numbers in bold indicate a significant difference ($p<0.05$).

Table S3. Differences in variables between pre-COVID19 pandemic and during COVID-19 pandemic by ratio of ORs among rheumatoid arthritis and osteoarthritis patients, weighted% (95% CI), in the data obtained from the KNHANES

Variables		Overall (1998-2021)	Pre-COVID-19 pandemic (1998- 2019)	COVID-19 pandemic (2020 and 2021)	Ratio of OR (95% CI)
		Weighted OR(95%CI)	Weighted OR(95%CI)	Weighted OR(95%CI)	
RA and OA					
Age	19-59	1.000 (reference)	1.000 (reference)	1.000 (reference)	1.000 (reference)
	≥60	**8.306 (7.959 to 8.668)**	**8.317 (7.957 to 8.693)**	**9.627 (8.148 to 11.375)**	1.158 (0.974 to 1.376)
Sex	Female	1.000 (reference)	1.000 (reference)	1.000 (reference)	1.000 (reference)
	Male	**0.263 (0.251 to 0.274)**	**0.260 (0.248 to 0.272)**	**0.294 (0.253 to 0.342)**	1.131 (0.966 to 1.324)
Region	Urban	1.000 (reference)	1.000 (reference)	1.000 (reference)	1.000 (reference)
	Rural	**1.829 (1.716 to 1.949)**	**1.866 (1.747 to 1.994)**	**1.492 (1.188 to 1.875)**	0.800 (0.631 to 1.015)
Education	High school or less	1.000 (reference)	1.000 (reference)	1.000 (reference)	1.000 (reference)
	College or more	**0.132 (0.124 to 0.140)**	**0.126 (0.118 to 0.135)**	**0.162 (0.137 to 0.192)**	**1.286 (1.072 to 1.542)**

Income	High	1.000 (reference)	1.000 (reference)	1.000 (reference)	1.000 (reference)
	Low	**2.692 (2.582 to 2.807)**	**2.669 (2.554 to 2.789)**	**2.935 (2.559 to 3.367)**	1.100 (0.952 to 1.270)

Abbreviations: CI, confidence interval; KNHANES, Korea National Health and Nutrition Examination Survey; OA, osteoarthritis; OR, odds ratio; RA, rheumatoid arthritis.

The numbers in bold indicate a significant difference ($p < 0.05$).
